# (*E*)-*N*′-[(2-Hy­droxy­naphthalen-1-yl)methyl­idene]-4-methyl­benzohydrazide

**DOI:** 10.1107/S160053681101035X

**Published:** 2011-03-26

**Authors:** Shi-Yong Liu, Shan-Shan Sun, Ting-Ting Zheng, Xiang-Lei Zheng, Xiao-Feng Zhao, Xiao-Fang Li

**Affiliations:** a College of Chemistry & Pharmacy, Taizhou University, Taizhou Zhejiang 317000, People’s Republic of China; b Department of Chemistry, Liaoning Normal University, Dalian 116029, People’s Republic of China

## Abstract

In the title compound, C_19_H_16_N_2_O_2_, the benzene ring and the naphthyl ring system form a dihedral angle of 8.7 (3)° and an intra­molecular O—H⋯N hydrogen bond generates an *S*(6) ring. In the crystal, mol­ecules are linked by N—H⋯O hydrogen bonds, forming *C*(4) chains propagating in [001].

## Related literature

For hydrazones we have reported previously and background references, see: Liu & You (2010*a* ▶,*b* ▶,*c* ▶); Liu & Wang (2010*a* ▶,*b* ▶). For a related structure, see: Cao (2009 ▶).
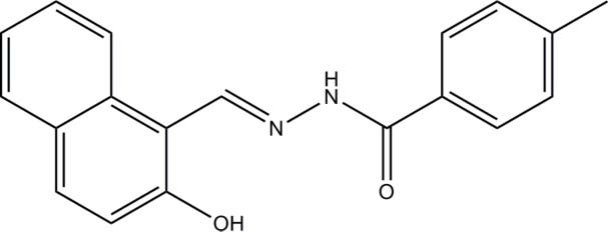


## Experimental

### 

#### Crystal data

C_19_H_16_N_2_O_2_
*M* *_r_* = 304.34
Monoclinic, 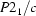

*a* = 11.014 (2) Å
*b* = 15.487 (2) Å
*c* = 9.150 (1) Å
β = 93.503 (3)°
*V* = 1557.8 (4) Å^3^
*Z* = 4
Mo *K*α radiation
μ = 0.09 mm^−1^
*T* = 298 K
0.20 × 0.17 × 0.15 mm

#### Data collection

Bruker SMART CCD diffractometer
Absorption correction: multi-scan (*SADABS*; Bruker, 2001 ▶) *T*_min_ = 0.983, *T*_max_ = 0.987
12464 measured reflections
3335 independent reflections
1515 reflections with *I* > 2σ(*I*)
*R*_int_ = 0.065

#### Refinement

*R*[*F* ^2^ > 2σ(*F* ^2^)] = 0.068
*wR*(*F* ^2^) = 0.192
*S* = 0.93
3335 reflections
213 parameters
1 restraint
H atoms treated by a mixture of independent and constrained refinement
Δρ_max_ = 0.16 e Å^−3^
Δρ_min_ = −0.25 e Å^−3^

### 

Data collection: *SMART* (Bruker, 2007 ▶); cell refinement: *SAINT* (Bruker, 2007 ▶); data reduction: *SAINT*; program(s) used to solve structure: *SHELXTL* (Sheldrick, 2008 ▶); program(s) used to refine structure: *SHELXTL*; molecular graphics: *SHELXTL*; software used to prepare material for publication: *SHELXTL*.

## Supplementary Material

Crystal structure: contains datablocks global, I. DOI: 10.1107/S160053681101035X/hb5818sup1.cif

Structure factors: contains datablocks I. DOI: 10.1107/S160053681101035X/hb5818Isup2.hkl

Additional supplementary materials:  crystallographic information; 3D view; checkCIF report

## Figures and Tables

**Table 1:** Hydrogen-bond geometry (Å, °)

*D*—H⋯*A*	*D*—H	H⋯*A*	*D*⋯*A*	*D*—H⋯*A*
N2—H2⋯O2^i^	0.90 (1)	2.02 (1)	2.897 (3)	164 (3)
O1—H1⋯N1	0.82	1.86	2.586 (3)	146

Symmetry code: (i) 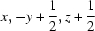.

**Fig. 1.**
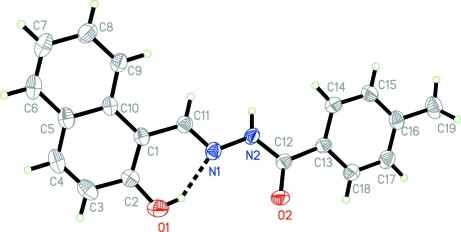
The molecular structure of the title compound. Displacement ellipsoids are drawn at the 30% probability level. Hydrogen atoms are shown as spheres of arbitrary radius and the intramolecular hydrogen bond is drawn as a dashed line.

**Fig. 2.**
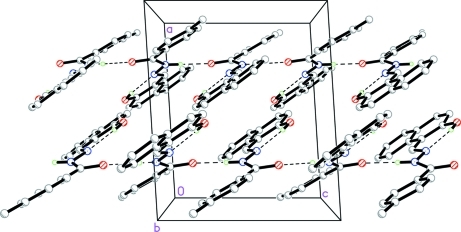
The packing of the title compound, viewed along the b axis. Hydrogen bonds are shown as dashed lines. Hydrogen atoms not involved in hydrogen bonding have been omitted.

## supplementary crystallographic information

### Comment

As a continuation of our structural studies of hydrazones (Liu & You, 2010*a*,*b*,*c*; Liu & Wang, 2010*a*,*b*), we report herein the crystal structure of the title compound, (I) (Fig. 1).

The dihedral angle between the C1—C10 benzene ring and the C13—C18 naphthyl ring is 8.7 (3)°. All the bond lengths are comparable to those observed in related structures (Cao, 2009) and those we reported previously.

In the crystal structure, molecules are linked through N–H···O hydrogen bonds, to form one-dimensional chains running along the *c* axis (Fig. 2 and Table 1).

### Experimental

The title compound was prepared by the condensation reaction of 2-hydroxy-1-naphthaldehyde (0.05 mol, 8.6 g) and 4-methylbenzohydrazide (0.05 mol, 7.5 g) in anhydrous methanol (200 ml) at ambient temperature. Colourless blocks were obtained by slow evaporation of the solution for a period of a week.

### Refinement

H2 was located from a difference Fourier map and refined isotropically, with the N–H distance restrained to 0.90 (1) Å. The remaining H atoms were positioned geometrically and constrained to ride on their parent atoms, with C–H distances of 0.93–0.96 Å, O–H distance of 0.82 Å, and with *U*_iso_(H) = 1.2*U*_eq_(C) and 1.5*U*_eq_(O and C19).

### Crystal data

**Table:**

C_19_H_16_N_2_O_2_	*F*(000) = 640
*M**_r_* = 304.34	*D*_x_ = 1.298 Mg m^−^^3^
Monoclinic, *P*2_1_/*c*	Mo *K*α radiation, λ = 0.71073 Å
Hall symbol: -P 2ybc	Cell parameters from 1270 reflections
*a* = 11.014 (2) Å	θ = 2.3–26.4°
*b* = 15.487 (2) Å	µ = 0.09 mm^−^^1^
*c* = 9.150 (1) Å	*T* = 298 K
β = 93.503 (3)°	Block, colourless
*V* = 1557.8 (4) Å^3^	0.20 × 0.17 × 0.15 mm
*Z* = 4	

### Data collection

**Table:**

Bruker SMART CCD diffractometer	3335 independent reflections
Radiation source: fine-focus sealed tube	1515 reflections with *I* > 2σ(*I*)
graphite	*R*_int_ = 0.065
ω scans	θ_max_ = 27.0°, θ_min_ = 1.9°
Absorption correction: multi-scan (*SADABS*; Bruker, 2001)	*h* = −14→13
*T*_min_ = 0.983, *T*_max_ = 0.987	*k* = −19→19
12464 measured reflections	*l* = −11→11

### Refinement

**Table:**

Refinement on *F*^2^	Primary atom site location: structure-invariant direct methods
Least-squares matrix: full	Secondary atom site location: difference Fourier map
*R*[*F*^2^ > 2σ(*F*^2^)] = 0.068	Hydrogen site location: inferred from neighbouring sites
*wR*(*F*^2^) = 0.192	H atoms treated by a mixture of independent and constrained refinement
*S* = 0.93	*w* = 1/[σ^2^(*F*_o_^2^) + (0.0921*P*)^2^] where *P* = (*F*_o_^2^ + 2*F*_c_^2^)/3
3335 reflections	(Δ/σ)_max_ = 0.001
213 parameters	Δρ_max_ = 0.16 e Å^−^^3^
1 restraint	Δρ_min_ = −0.25 e Å^−^^3^

### Special details

**Table:**

Geometry. All esds (except the esd in the dihedral angle between two l.s. planes) are estimated using the full covariance matrix. The cell esds are taken into account individually in the estimation of esds in distances, angles and torsion angles; correlations between esds in cell parameters are only used when they are defined by crystal symmetry. An approximate (isotropic) treatment of cell esds is used for estimating esds involving l.s. planes.
Refinement. Refinement of F^2^ against ALL reflections. The weighted R-factor wR and goodness of fit S are based on F^2^, conventional R-factors R are based on F, with F set to zero for negative F^2^. The threshold expression of F^2^ > 2sigma(F^2^) is used only for calculating R-factors(gt) etc. and is not relevant to the choice of reflections for refinement. R-factors based on F^2^ are statistically about twice as large as those based on F, and R- factors based on ALL data will be even larger.

### Fractional atomic coordinates and isotropic or equivalent isotropic displacement parameters (Å^2^)

**Table:**

	*x*	*y*	*z*	*U*_iso_*/*U*_eq_	
N1	0.6996 (2)	0.17460 (14)	−0.0689 (2)	0.0519 (6)	
N2	0.7488 (2)	0.24189 (14)	0.0157 (2)	0.0525 (7)	
O1	0.5593 (2)	0.11437 (14)	−0.2818 (2)	0.0706 (7)	
H1	0.5958	0.1516	−0.2329	0.106*	
O2	0.75910 (18)	0.32363 (11)	−0.1872 (2)	0.0596 (6)	
C1	0.6450 (2)	0.02635 (17)	−0.0848 (3)	0.0472 (7)	
C2	0.5756 (3)	0.03725 (19)	−0.2161 (3)	0.0553 (8)	
C3	0.5169 (3)	−0.0341 (2)	−0.2832 (4)	0.0706 (9)	
H3	0.4679	−0.0261	−0.3686	0.085*	
C4	0.5303 (3)	−0.1141 (2)	−0.2263 (4)	0.0702 (10)	
H4	0.4897	−0.1600	−0.2731	0.084*	
C5	0.6039 (3)	−0.13015 (19)	−0.0983 (4)	0.0586 (8)	
C6	0.6208 (3)	−0.2140 (2)	−0.0396 (4)	0.0693 (10)	
H6	0.5810	−0.2604	−0.0857	0.083*	
C7	0.6934 (3)	−0.2278 (2)	0.0816 (4)	0.0761 (10)	
H7	0.7041	−0.2835	0.1183	0.091*	
C8	0.7527 (3)	−0.1584 (2)	0.1524 (4)	0.0721 (10)	
H8	0.8030	−0.1682	0.2361	0.087*	
C9	0.7375 (3)	−0.07684 (18)	0.1001 (3)	0.0575 (8)	
H9	0.7780	−0.0316	0.1489	0.069*	
C10	0.6618 (2)	−0.05878 (17)	−0.0264 (3)	0.0480 (7)	
C11	0.6973 (2)	0.10041 (17)	−0.0080 (3)	0.0494 (7)	
H11	0.7299	0.0939	0.0876	0.059*	
C12	0.7759 (2)	0.31563 (17)	−0.0533 (3)	0.0464 (7)	
C13	0.8249 (2)	0.38767 (17)	0.0401 (3)	0.0456 (7)	
C14	0.8877 (3)	0.37590 (19)	0.1745 (3)	0.0568 (8)	
H14	0.9016	0.3205	0.2110	0.068*	
C15	0.9292 (3)	0.4465 (2)	0.2537 (3)	0.0639 (9)	
H15	0.9706	0.4374	0.3440	0.077*	
C16	0.9125 (3)	0.5301 (2)	0.2056 (3)	0.0579 (8)	
C17	0.8512 (3)	0.54064 (19)	0.0705 (4)	0.0626 (9)	
H17	0.8382	0.5961	0.0340	0.075*	
C18	0.8091 (3)	0.47147 (18)	−0.0113 (3)	0.0577 (8)	
H18	0.7694	0.4809	−0.1026	0.069*	
C19	0.9554 (3)	0.6073 (2)	0.2941 (4)	0.0829 (11)	
H19A	1.0096	0.6410	0.2388	0.124*	
H19B	0.9973	0.5883	0.3834	0.124*	
H19C	0.8867	0.6419	0.3165	0.124*	
H2	0.752 (3)	0.2325 (19)	0.1132 (12)	0.080*	

### Atomic displacement parameters (Å^2^)

**Table:**

	*U*^11^	*U*^22^	*U*^33^	*U*^12^	*U*^13^	*U*^23^
N1	0.0629 (16)	0.0419 (13)	0.0516 (15)	−0.0017 (12)	0.0087 (12)	−0.0052 (12)
N2	0.0786 (18)	0.0367 (13)	0.0428 (14)	−0.0055 (12)	0.0079 (13)	−0.0006 (11)
O1	0.0734 (16)	0.0762 (15)	0.0610 (15)	0.0081 (13)	−0.0060 (12)	0.0068 (12)
O2	0.0847 (16)	0.0572 (13)	0.0364 (12)	−0.0029 (10)	−0.0010 (10)	0.0036 (9)
C1	0.0443 (17)	0.0479 (17)	0.0500 (18)	−0.0007 (13)	0.0075 (14)	−0.0086 (13)
C2	0.0518 (18)	0.0588 (19)	0.055 (2)	0.0048 (15)	0.0027 (16)	−0.0034 (16)
C3	0.052 (2)	0.094 (3)	0.065 (2)	−0.0017 (19)	−0.0027 (17)	−0.019 (2)
C4	0.058 (2)	0.069 (2)	0.084 (3)	−0.0140 (18)	0.0074 (19)	−0.029 (2)
C5	0.0523 (19)	0.057 (2)	0.067 (2)	−0.0060 (15)	0.0136 (17)	−0.0147 (17)
C6	0.074 (2)	0.049 (2)	0.087 (3)	−0.0107 (17)	0.023 (2)	−0.0150 (18)
C7	0.094 (3)	0.0457 (19)	0.091 (3)	−0.0033 (19)	0.025 (2)	0.0035 (19)
C8	0.088 (3)	0.058 (2)	0.071 (2)	0.0006 (18)	0.0055 (19)	0.0081 (17)
C9	0.070 (2)	0.0435 (17)	0.060 (2)	−0.0018 (15)	0.0105 (17)	0.0001 (15)
C10	0.0438 (17)	0.0467 (17)	0.0544 (19)	−0.0039 (13)	0.0095 (14)	−0.0081 (14)
C11	0.0538 (18)	0.0454 (17)	0.0495 (17)	0.0011 (14)	0.0065 (14)	−0.0012 (14)
C12	0.0497 (17)	0.0426 (16)	0.0471 (17)	0.0046 (13)	0.0054 (13)	0.0005 (14)
C13	0.0489 (17)	0.0470 (16)	0.0414 (16)	0.0023 (13)	0.0063 (13)	0.0020 (13)
C14	0.0601 (19)	0.0570 (19)	0.0528 (19)	−0.0048 (15)	−0.0001 (16)	0.0112 (15)
C15	0.067 (2)	0.074 (2)	0.0491 (19)	−0.0151 (18)	−0.0055 (16)	0.0005 (17)
C16	0.0520 (18)	0.063 (2)	0.059 (2)	−0.0116 (15)	0.0114 (16)	−0.0079 (16)
C17	0.074 (2)	0.0442 (17)	0.070 (2)	−0.0008 (15)	0.0066 (18)	−0.0003 (16)
C18	0.066 (2)	0.0485 (18)	0.058 (2)	−0.0002 (15)	−0.0016 (16)	0.0042 (15)
C19	0.089 (3)	0.084 (2)	0.077 (2)	−0.025 (2)	0.012 (2)	−0.024 (2)

### Geometric parameters (Å, °)

**Table:**

N1—C11	1.278 (3)	C8—C9	1.358 (4)
N1—N2	1.388 (3)	C8—H8	0.9300
N2—C12	1.347 (3)	C9—C10	1.413 (4)
N2—H2	0.902 (10)	C9—H9	0.9300
O1—C2	1.344 (3)	C11—H11	0.9300
O1—H1	0.8200	C12—C13	1.486 (4)
O2—C12	1.234 (3)	C13—C14	1.386 (4)
C1—C2	1.394 (4)	C13—C18	1.388 (3)
C1—C10	1.430 (4)	C14—C15	1.375 (4)
C1—C11	1.446 (3)	C14—H14	0.9300
C2—C3	1.403 (4)	C15—C16	1.376 (4)
C3—C4	1.348 (4)	C15—H15	0.9300
C3—H3	0.9300	C16—C17	1.382 (4)
C4—C5	1.405 (4)	C16—C19	1.504 (4)
C4—H4	0.9300	C17—C18	1.371 (4)
C5—C6	1.413 (4)	C17—H17	0.9300
C5—C10	1.418 (4)	C18—H18	0.9300
C6—C7	1.344 (4)	C19—H19A	0.9600
C6—H6	0.9300	C19—H19B	0.9600
C7—C8	1.395 (4)	C19—H19C	0.9600
C7—H7	0.9300		
			
C11—N1—N2	116.7 (2)	C9—C10—C1	123.0 (2)
C12—N2—N1	117.7 (2)	C5—C10—C1	120.1 (3)
C12—N2—H2	127.2 (19)	N1—C11—C1	121.3 (3)
N1—N2—H2	114.8 (19)	N1—C11—H11	119.4
C2—O1—H1	109.5	C1—C11—H11	119.4
C2—C1—C10	119.0 (3)	O2—C12—N2	121.7 (3)
C2—C1—C11	120.3 (3)	O2—C12—C13	121.5 (2)
C10—C1—C11	120.7 (3)	N2—C12—C13	116.8 (2)
O1—C2—C1	123.0 (3)	C14—C13—C18	118.0 (3)
O1—C2—C3	117.3 (3)	C14—C13—C12	123.7 (2)
C1—C2—C3	119.7 (3)	C18—C13—C12	118.3 (3)
C4—C3—C2	121.2 (3)	C15—C14—C13	119.7 (3)
C4—C3—H3	119.4	C15—C14—H14	120.2
C2—C3—H3	119.4	C13—C14—H14	120.2
C3—C4—C5	121.9 (3)	C14—C15—C16	123.1 (3)
C3—C4—H4	119.1	C14—C15—H15	118.4
C5—C4—H4	119.1	C16—C15—H15	118.4
C4—C5—C6	122.4 (3)	C15—C16—C17	116.5 (3)
C4—C5—C10	118.0 (3)	C15—C16—C19	123.0 (3)
C6—C5—C10	119.7 (3)	C17—C16—C19	120.5 (3)
C7—C6—C5	121.2 (3)	C18—C17—C16	121.8 (3)
C7—C6—H6	119.4	C18—C17—H17	119.1
C5—C6—H6	119.4	C16—C17—H17	119.1
C6—C7—C8	119.9 (3)	C17—C18—C13	121.0 (3)
C6—C7—H7	120.1	C17—C18—H18	119.5
C8—C7—H7	120.1	C13—C18—H18	119.5
C9—C8—C7	120.6 (3)	C16—C19—H19A	109.5
C9—C8—H8	119.7	C16—C19—H19B	109.5
C7—C8—H8	119.7	H19A—C19—H19B	109.5
C8—C9—C10	121.8 (3)	C16—C19—H19C	109.5
C8—C9—H9	119.1	H19A—C19—H19C	109.5
C10—C9—H9	119.1	H19B—C19—H19C	109.5
C9—C10—C5	116.9 (3)		

### Hydrogen-bond geometry (Å, °)

**Table:**

*D*—H···*A*	*D*—H	H···*A*	*D*···*A*	*D*—H···*A*
N2—H2···O2^i^	0.90 (1)	2.02 (1)	2.897 (3)	164 (3)
O1—H1···N1	0.82	1.86	2.586 (3)	146

Symmetry codes: (i) *x*, −*y*+1/2, *z*+1/2.
